# Enhanced electromechanical performance of Si-modified lead-free BiFeO_3_-BaTiO_3_ ceramics for high-temperature piezoelectric applications

**DOI:** 10.1371/journal.pone.0318768

**Published:** 2025-03-07

**Authors:** Hussein Alrobei, Muhammad Habib, Shoaib Ali, Rizwan Ahmed Malik, Muhammad Javid Iqbal, Qamar Iqbal, Fadl A. Essa, Z.M. Omara

**Affiliations:** 1 Department of Mechanical Engineering, College of Engineering, Prince Sattam bin Abdulaziz University, Al-Kharj, 11942, Saudi Arabia; 2 Department of Physics, University of Azad Jammu and Kashmir, Muzaffarabad, Azad Kashmir, 13100, Pakistan; 3 Department of Physics, University of Peshawar, 25120, Peshawar, Pakistan; 4 Department of Metallurgy and Materials Engineering, Faculty of Mechanical and Aeronautical Engineering, University of Engineering and Technology, 47050, Taxila, Pakistan; 5 Department of Physics, Riphah International University, 44000, Islamabad, Pakistan; 6 Mechanical Engineering Department, Faculty of Engineering, Kafrelsheikh University, Kafrelsheikh, 33516, Egypt; Zhejiang University, CHINA

## Abstract

Environmental pollution generated by industrial wastes are deteriorating land, water, and marine life, which raises major concerns about climate change. Since environmentally friendly piezoelectric materials can generate clean energy by applying mechanical forces, they are seen as viable agents for industrial applications. In recent research work, the Si-modified 0.70Bi_1.03_FeO_3_-0.30BaTiO_3_ (BF30BT) environmentally friendly piezoceramics were synthesized using a solid-state method followed by a thermal quenching process. The crystalline structure, microstructure, and electromechanical characteristics were explored as a function of Si for both dopants (BC; before calcination) and additives (AC; after calcination). The result of pure BF30BT ceramic reveals a dominant rhombohedral phase exhibiting a *d*_33_ of 251 pC/N with a higher *T*_C_ of 560 °C. The Si-doping gradually transformed the predominant rhombohedral phase to the rhombohedral-tetragonal mixed phase asymmetry as a result a good balance was achieved among *d*_33_ (209 pC/N), *Q*_m_ (32.6), and k_p_ (0.32%) with a high *T*_C_ (465 °C). A giant-induced electric field bipolar strain of 0.39% corresponding to a large-signal piezoelectric coefficient *d*_33_^*^ ≈  750 pm/V was perceived in Si-doped BF30BT ceramic. The defect dipoles by acceptor doping play an essential role in the enhancement of piezoelectricity. The defect dipole aligns in the spontaneous polarization and also offers restoring force for domain switching leading to high asymmetric electrostrain. This study provides a good design benchmark for a new generation of eco-friendly large-strain actuator piezoceramics.

## 1. Introduction

**Fig 1:**
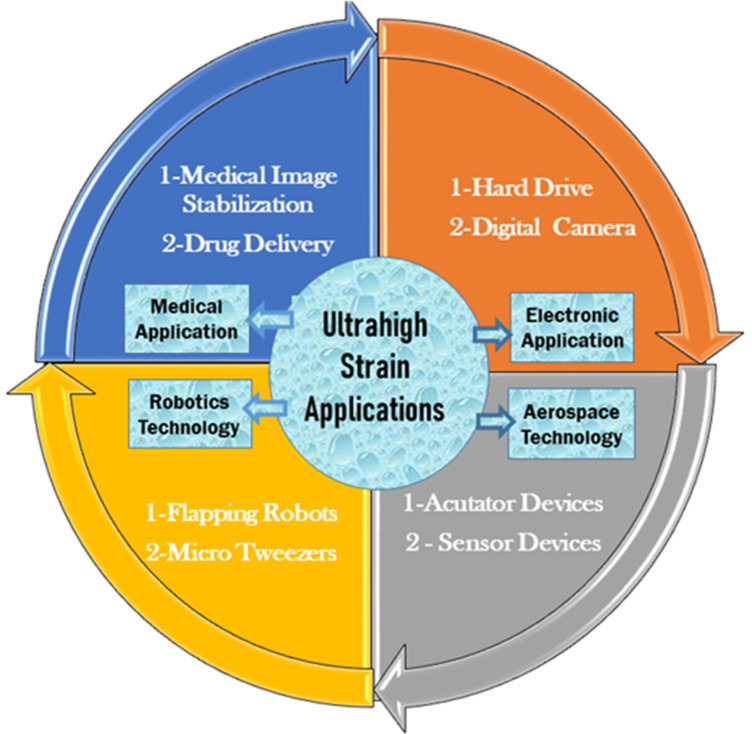
The potential applications of ultrahigh electrostrain piezoceramic materials and their preferred devices.

The concept of piezoelectric materials is the interconversion of mechanical stress and electrical voltage [1]. Piezoelectric ceramics are now widely used in electromechanical devices owing to their rapid response. The potential applications of high electrostrain piezoceramic materials are presented schematically in Fig 1. In general, piezoelectric materials can be categorized into two types such as lead-based and lead-free piezoceramics. Lead-based (Pb Zr_1-*x*_Ti_*x*_ O_3_, PZT) piezoelectric materials dominate the commercial market due to their superior piezoelectric performance and easily modifiable electric characteristics with simple fabrication. However, the high amount of lead (more than 60%) in the solid solution is a massive concern for the environment and human health. Therefore, significant attention has been devoted to materials, such as (Bi_0.5_Na_0.5_)TiO_3_ (BNT), (K_0.5_Na_0.5_)NbO_3_ (KNN), BaTiO_3_ BiFeO_3_ materials [2–4]. BNT-based materials reflect superior strain performance (0.4% – 0.7%) among these lead-free piezoelectric materials [5,6]. Unfortunately, the BNT piezoelectric ceramics possess high strain hysteresis (70%), low depolarization temperature ( < 120 °C), and a high electric field (80-100 kV/cm) is required for large electrostrain [7].

Now for real applications high strain under the low electric field is a significant challenge. Saito achieved a high piezoelectric constant(*d*_33_*) of 750 pm/V in textured ceramics under an ultralow electric field of 20 kV/cm [8]. However, the texturing process is complicated and expensive for mass production. Furthermore, a significant recoverable electro-strain 0.75% with *d*_33_^*^ of 3700 pm/V has been reported in the Fe^3+^-acceptor-doped BaTiO_3_ single crystals, utilizing the defect dipoles [9]. However, many parameters are required during the synthesis of a single crystal and also the large strain hysteresis (80%) of this work restricts their use for practical applications. Additionally, the BaTiO_3_ shows lower *T*_C_ and limits its use for high-temperature applications. Recently, the solid solution of BiFeO_3_-BaTiO_3_ (BF-BT) ceramics have gained papularity due to their good piezoelectric performance and high *T*_C_ ≥  450 °C [10]. However, the high leakage current and secondary phase formation in the BiFeO_3_-based material restrict its potential properties.

**Fig 2:**
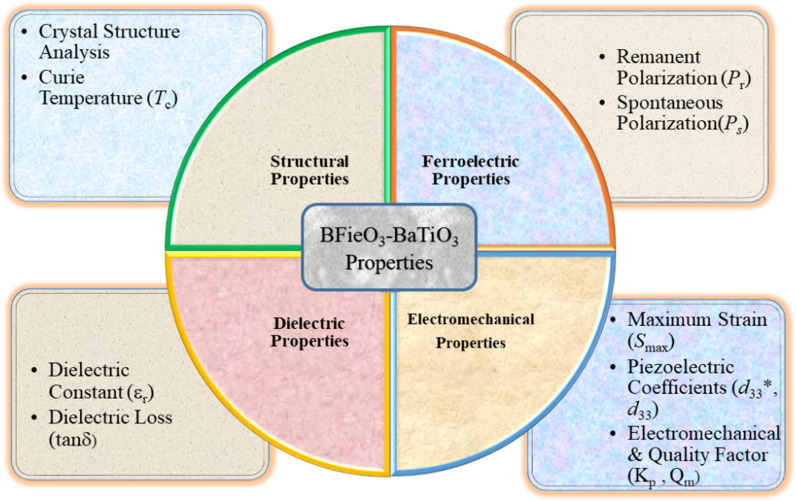
The functional properties of lead-free piezoelectric BiFeO_3_-BaTiO_3_ ceramic and desired parameters.

Many approaches have been adopted for the enhancement of electromechanical performance such as phase boundary engineering and local structure inhomogeneity or polar nano regions [11]. Defect dipole engineering is a novel material design strategy for high electrostrain performance. Recently ultrahigh electric field-induced strain of over 1% has been achieved in the acceptor-doped KNN-based [12,13]. Therefore, in this work, a new composition of acceptor doping is designed according to the chemical formula 0.7Bi_1.03_FeO_3_-0.3BaTi_1*-x*_Si_*x*_O_3_, where the Si^2+^ substitution on Ti^4+^ induces oxygen vacancy. This oxygen vacancy creates a defect dipole with A-site or B-site cation vacancy and the one-way round trip to this defect dipole under the bipolar applied field induces high asymmetric strain. The electromechanical properties of the lead-free piezoelectric BF-BT system are schematically studied as given in the schematic diagram of Fig 2.

## 2. Experimental procedure

### 2.1. Raw materials

The raw powders of the oxides and carbonates, such as Bi_2_O_3_ (99.99%, Sigma Aldrich), Si_2_O_3_ (99.99%, Alfa Aesar), Fe_2_O_3_ (≥ 99.99%, Sigma Aldrich), BaCO_3_ (≥ 99.99%, Sigma Aldrich) and TiO_2_ (≥ 99.95%, Sigma Aldrich) were used.

### 2.2. Material synthesis

A typical solid-state reaction technique was used to fabricate lead-free 0.70Bi_1.03_FeO_3_-0.30BaTi_1-*x*_Si_*x*_O_3_ ceramics with *x* =  0.00, 0.005(BC), 0.01(BC), 0.01(AC), where BC and AC show the addition of Si_2_O_3_ mol% powder before and after calcination, respectively. The 3mol% Bi_2_O_3_ excess powder was added for their compensation at high temperatures. The initial reagents were thoroughly mixed and ball-milled for 24 h in ethanol. Then, this slurry was dried at 120°C for 6 h and then calcined at 700 °C for 2 h at a heating rate of 10 °C/min. The calcined powder was re-milled for 6 hours to achieve chemical homogeneity. The 5 wt % polyvinyl alcohol (PVA) was added with calcinated powder and dried at 120 °C for at least 6 h. The dried powder was hand-grind by using a mortar and pestle. These crushed powders were passed from 100 µm mash and then, pressed into disk-shaped pellets with a ~10 mm diameter. To prevent Bi_2_O_3_ from volatilizing during the sintering process, pellets were buried in the same structural powder. Initially, the binder (PVA) was removed at 300°C for 1 h and then all samples were sintered for 3 h at a temperature of 980-1000 °C. All ceramics were immediately quenched in water to avoid the unstable temperature range and suppress non-ferroelectric secondary phases. The samples were polished to a thickness of 400 μm and silver paste was coated on both sides of the pellets and dried at 100 °C.

### 2.3 Characterization

X-ray diffraction (XRD, Bruker, D8 Advance) was used to examine the crystalline structures of the sintered samples. An impedance analyzer (HP4194A, Agilent) connected with a computer-programmed furnace system was used to find out the dielectric properties. The ceramics were poled in the silicone oil bath under 50 kV/cm for 30 min and their *d*_33_ values were measured by using the *d*_33_-meter (IACAS, ZJ-6B). A ferroelectric measuring device (aixACCT System GmbH 1000, Germany) used an electric field of 50 kV/cm to measure the ferroelectric and piezoelectric properties. The resonance-antiresonance measurements were made using an Agilent 4294A precision impedance analyzer to calculate mechanical quality factor (*Q*_m_) and planar electromechanical coupling coefficient (k_p_) (Hewlett-Packard, Palo Alto, CA).

## 3. Results and discussion

**Fig 3:**
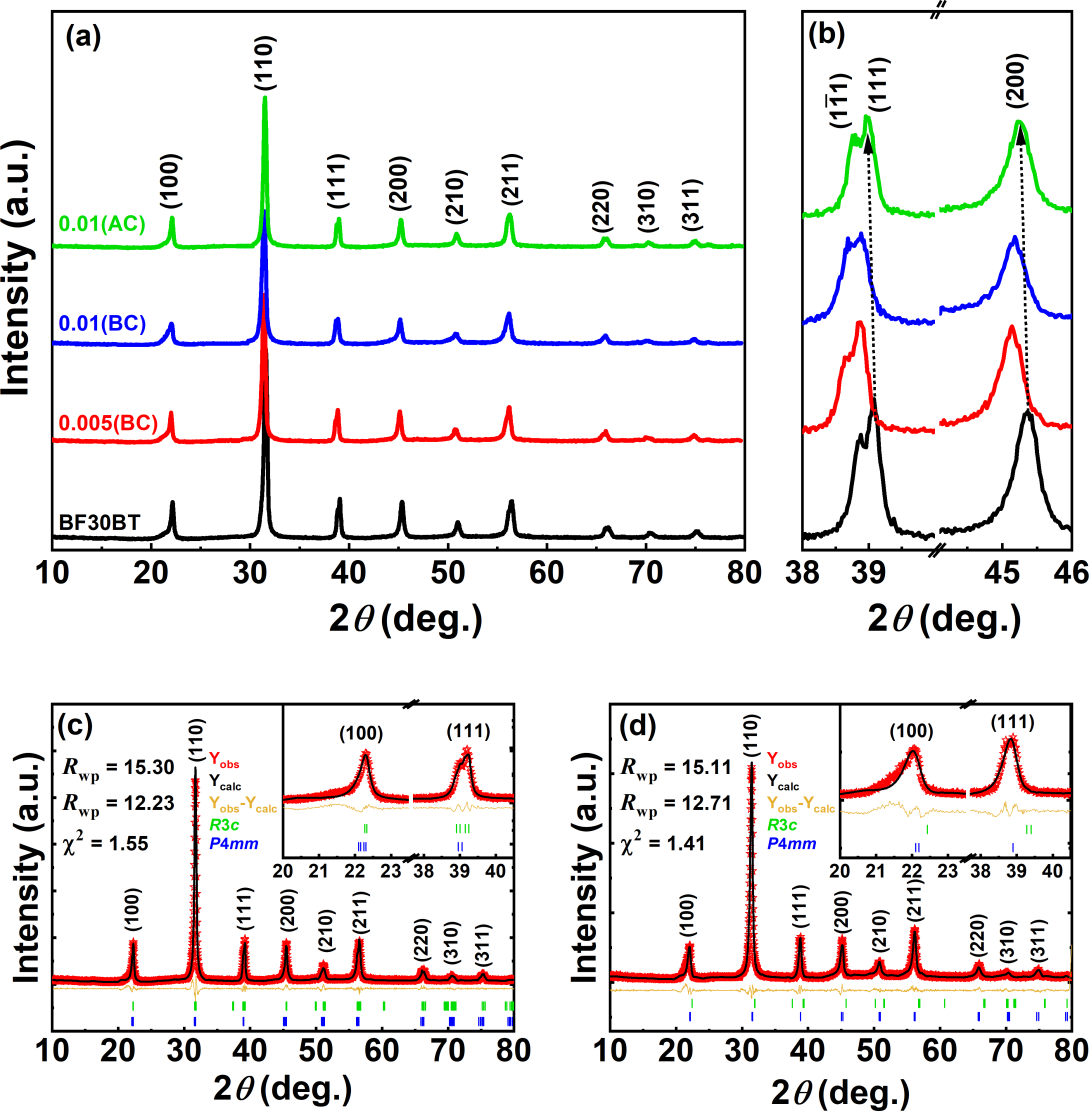
(a) The X-ray diffraction patterns in the suggested range of 2*θ* =  10° − 60°, (b) magnified view for (111) peak at 2*θ* =  38° − 40° and (200) peak at 2*θ* =  38° − 40°, and (c, d) XRD Rietveld refinements patterns of *x* =  0.00(BC), 0.01(BC) samples for Si-modified BF30BT solid solutions.

Fig 3(a) shows the XRD patterns for BF30BT sintered ceramics at ambient temperature in the 2*θ* range of 10° − 80° with *x* =  0.00(BC), 0.005(BC), 0.01(BC), 0.01(AC). All compositions demonstrate a single perovskite structure, indicating the formation of a stable solid solution. This indicates that the Si^3+^ ion has completely diffused in the BF30BT lattice and has led to a stable solid solution with a pure phase structure. In the rhombohedral (R) unit cell, the direction of polarization is (111), and for the tetragonal (T), it is either (100) or (200). The splitting of the (200) and (111) peaks allows the identification of the T and R phases [14,15]. Therefore, the magnified view of the (111) and (200) diffraction peaks are shown in Fig 3(b). A slight shift of the peak toward the lower angle is due to the larger ionic radius of Si^3+^ than the Ti^4+^ ion. The doublet (111)/(1$\bar{1}$1) peaks and a single (200) peak for undoped BF30BT indicating the predominance of R symmetry. However, after Si-doping the (1$\bar{1}$1) and (111) peaks gradually merged, suggesting the R+T mixed phase symmetry. On the other hand, for 0.01(AC) a distinct splitting can be observed near 39° suggesting that Si_2_O_3_ addition before calcination is more effective as compared to after calcination Si-doping. The MPB between the R and T phases was also previously reported for the BF-BT ceramics [16,17].

To analyze the exact crystal structure Rietveld refinement was performed for BF30BT and 0.01(BC) samples as shown in Fig 3(c, d). In the previous investigation of BF-BT solid solution the *R*3*c* and *P*4*mm* mixed-phase model have been applied. Therefore, in this work, a biphasic model (*R*3*c* and *P*4*mm*) was used during the refinement method which shows the best goodness of fit (χ^2^) values. The rhombohedral lattice constant *a*_R_ =  4.0100 Å was deduced from the hexagonal unit cell indirectly. The tetragonal lattice constant *a*_T_ =  3.9956 Å and *c*_T_ =  4.0171 Å, with tetragonality *c*_T_/*a*_T_ =  1.005 was found for pure BF30BT ceramic. By Si-doping with *x* =  0.01, *a*_R_ =  4.0117 Å, *a*_T_ =  3.9894 Å, *c*_T_ =  4.0222 Å, and *c*_T_/*a*_T_ =  1.012 were found. This large lattice distortion plays a significant role in the enhancement of piezoelectricity [18]. Therefore, the coexistence of R and T phases with the largest lattice distortion in BF30BTSi ceramic leads to a high piezoelectric response.

**Fig 4:**
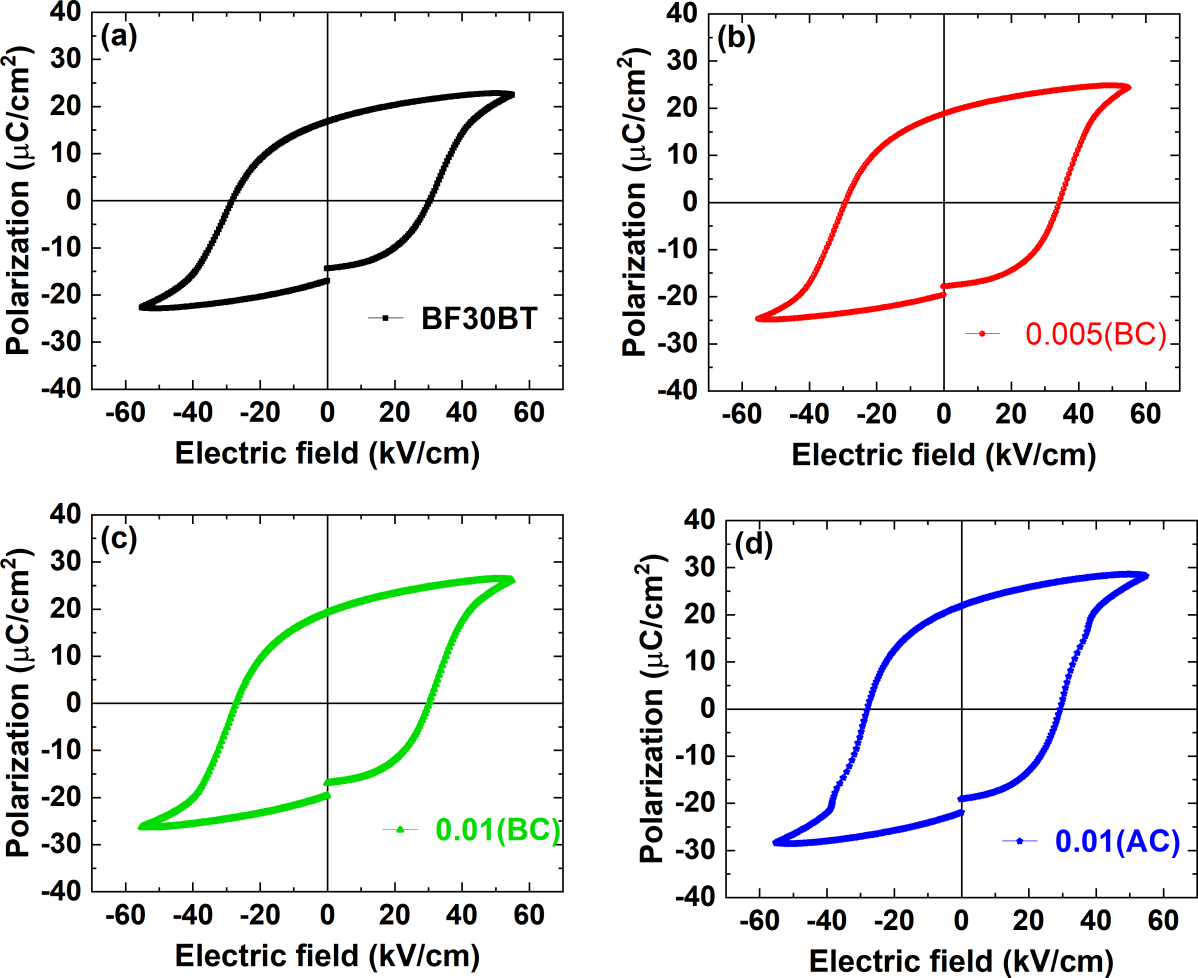
(a-d) Ferroelectric polarization (*P* − *E*) hysteresis loops for Si-doped BF30BT ceramics.

Fig 4(a-d) depicts the the ferroelectric polarization-electric field (*P*−*E*) hysteresis loops of the BF30BT ceramic with various Si^3+^ concentrations under the 55 kV/cm applied electric field at 10 Hz. Each loop is well-saturated and symmetrical, with sharp loop corners. The pure sample exhibits a hard *P*−*E* hysteresis loop, confirming the ferroelectric nature of BF30BT ceramics, and is associated with domain-wall pinning [19] resulting from the defects. The addition of Si has a considerable impact on the contour of the *P*−*E* loops and the polarization response of the BFBT system. At 0.00 ≤  *x* ≤  0.005(BC), typical ferroelectric polarization hysteresis loops were identified as *x* increased, demonstrating that adequate Si concentrations can increase domain-wall pinning and improve ferroelectric characteristics. When the applied electric field is removed, the ferroelectric phase’s long-range order maintains its polarization. Therefore, the pure BF30BT ceramic displayed the remnant polarization (*P*_r_) of 16.75 μC/cm^2^ and saturation polarization (*P*_s_) of 22.7 μC/cm^2^. The introduction of small amount of Si in to BF30BT ceramics slightly increased ferroelectric response and showed the highest *P*_r_ of 21.9 μC/cm^2^ and *P*_S_ of 29.3 μC/cm^2^ for *x* =  0.01(AC) near MBP composition.

**Fig 5:**
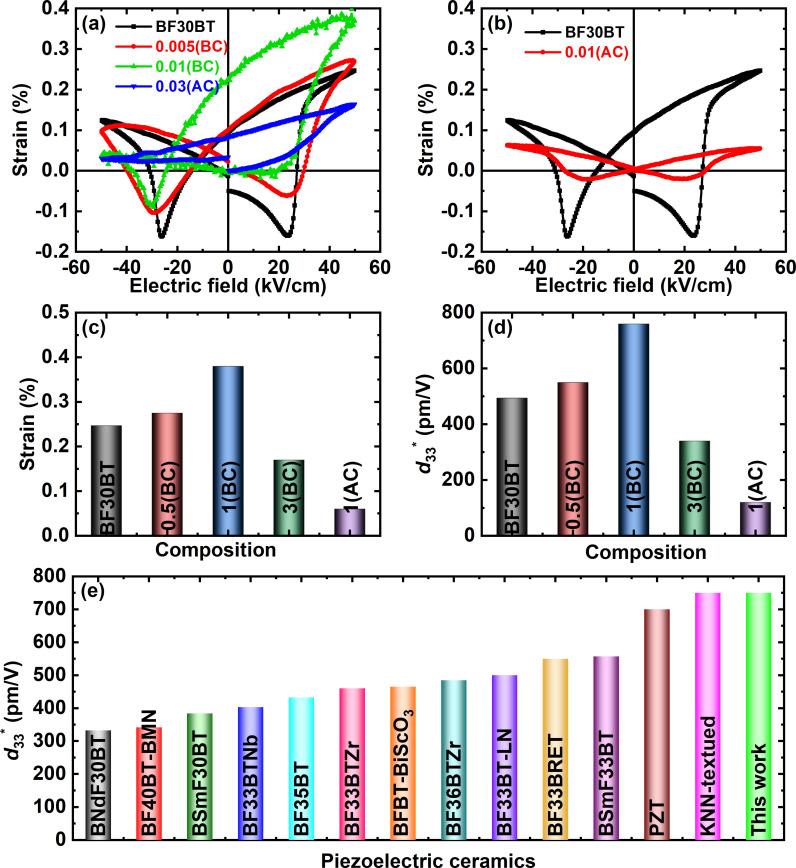
(a, b) Butterfly bipolar strain-electric field (*S* − *E*) curves recorded under an applied electric field of 50 kV/cm, (c) maximum strain (*S*_max_), and (d) *d*_33_, *d*_33_^ *^ of BF30BT ceramic as a function of Si^3 +^ concentration. (e) Nd-doped BF30BT [23], BF40BT-BiM_2/3_Nb_1/3_O_3_ (BF40BT-BMN)[57], Sm-doped BF30BT (BSmF30BT) [24], Nb-doped BF33BT (BF33BTNb) [1], BF35BT [58], Zr-doped BF33BT (BF33BZrT) [14], BFBT-BiScO_3_ [28], Zr-doped BF36BT [30], LiNbO_3_-BF33BT (BF33BT-LN) [31], Ba-site Sm-doped BF33BT (BF33BSmT) [59], Bi-site BF33BT (BSmF33BT) [60], PZT and KNN-textured [8].

In order to investiage the effect of aceeptor doping on the defect chemistry and origin of high piezoelectric performance the bipolar piezoelectric strain-electric field (*S*−*E*) curves were measured under the 50 kV/cm as shown in Fig 5(a,b). Actually, the acceptor doping are induces $V_{{\text{O}}^{2-}}^{••}$ for charge balance and form defect dipoles [20]. Recently, asymmetric *S*−*E* loops with a large strain were achieved in the lead-free ceramics by creating defect dipole [21,22]. For the sample Si-doping before calcination the maximum electrostrain (*S*_max_) value of 0.39% is achtived at 50 kV/cm for the 0.5(BC) as shown in Fig 5c. The activation energy of $V_{{\text{O}}^{2-}}^{••}$hopping (about 1 eV) is substantially larger than the activation energy of domain barriers (roughly 0.1 eV) [4]. The one way trip of this defect dipole unfer the bipolar electric field produces asymmetric *S*−*E* hysteresis loop [12]. Fig 5(d) illustrates the computed *d*_33_* values as a function of the Si concentration. A highest of 750 pm/V was achieved for *x* =  0.01(BC) composition which exceeds to reported lead-free ceramics and many lead-free BF-BT ceramics and equivalent to KNN-textured materials as shown Fig 5(e). More detailed is given in Table 1 for comparasion the piezoelectric strain response under the applied electric field and its working temperature range.

**Table 1:** Summary of applied field (*E*_a_), Curie temperature (*T*_C_) and dynamic piezoelectric constant (*d*_33_^*^) and comparasion with other piezoelectric ceramics.

Composition	*E*_*a*_ (kV/cm)	*T*_C_ (°C)	*d*_33_^*^ (pm/V)	Ref.
BNdF30BT	60	380	333	[23]
BF33BT	55	476	352	[10]
BF30BT-Sm	80	341	384	[24]
BF35BT		415	405	[25]
BF30BT-*x*Bi(Zn_2/3_Nb_1/3_)O_3_	100	480	424	[26]
BF33BLa*x*T-	55	482	458	[27]
BFBT-BiMe	50	400	465	[28]
BF33BT-BiGaO_3_	55	454	471	[29]
BF36BT-*x*Zr	60	355	485	[30]
BLa*x*F30BT	55	360	500	[18]
BF33BT-*x*LiNbO_3_	35	390	500	[31]
BF37BT-*x*Bi(Mg_2/3_Nb_1/3_)O_3_	100	390	544	[32]
BF*x*BSmT	55	450	552	[33]
BLaF33BT	55	336	570	[15]
BF40BT-*x*Bi(Zn_0.5_Ti_0.5_)O_3_	40	330	600	[34]
BF36BT	60	450	633	[35]
BF33BT-*x*(Ba_0.8_Ca_0.2_)ZrO_3_	60	380	640	[36]
CZ5	40	192	320	[37]
KNNS-SZ-BNZ	40	250	370	[38]
KNN-BLT-BZ	40	250	400	[39]
KNN-BLT-BZ-Mn	40	243	475	[40]
KNNS-NKZS	20	240	480	[41]
KNNTS_*x*_-BSNZ	30	280	497	[42]
KNN-BSNZ		268	508	[43]
KNNSb-BZ-BNH	20	180	600	[44]
KNN-BNAZZ	45	239	608	[45]
PZST	50	160	672	[46]
PZT4	20	250	700	[8]
Si-doped BF30BT	50	460	750	This work

BiFeO_3_-BaTiO_3_(BF-BT) [10,15,18,23–36], (K,Na)NbO_3_ (KNN) [37–45] and Pb(Zr,Ti)O_3_ (PZT) [8,46].

This significant increase in bipolar strain may be ascribed to the phase transition from ferroelectrics to relaxor ferroelectrics and defect-dipole. The negative strain (*S*_neg_) indicates the non-180° reorientation of domains, and positive strain is the sum of intrinsic piezoelectric lattice strain and extrinsic reversible domain switching strain [47]. A dramatic decrease occurred in the |−*S*_neg_| ≈ 0.18% of the pure BF30BT to 0.01% for Si-doped ceramic at *x* =  0.03(AC). This low *S*_neg_ value shows a decrease in hard ferroelectric behavior with Si-acceptor doping. The higher piezoelectric strain performance for the optimum composition may be attributed to the dominating relaxor phase, dominance of extrinsic contribution from the reversible electric field-induced phase shit and defect-dipole induced asymmetric strain [2,3,48]. Nevertheless, the bipolar *S*−*E* curves change into sprout-shaped curves and the *S*_neg_ value decreases to 0.01 percent at *x* =  0.03(AC). With Si-doping the drop in *S*_neg_ values provides evidence for the relaxor ferroelectric effect. Therefore, the Si-addition before calcination is a more effective way to the improvement of electromechanical performance as compared to the after calcination. In the current study, slight asymmetry in the polarization hysteresis loops were also observed, although less pronounced than the strain hysteresis loops. This difference may arise because the electrostrain and the polarization hysteresis loops were recorded under different frequencies. Its further indicates that asymmetric behavior is more pronounced at lower frequencies. This behavior also hints towards the presence of defect structures in the investigated compositions. These defect structures reconfigure more slowly under the applied field as compared to domain polarization reversal. This phenomenon is in good agreement with previously reported work [49].

**Fig 6:**
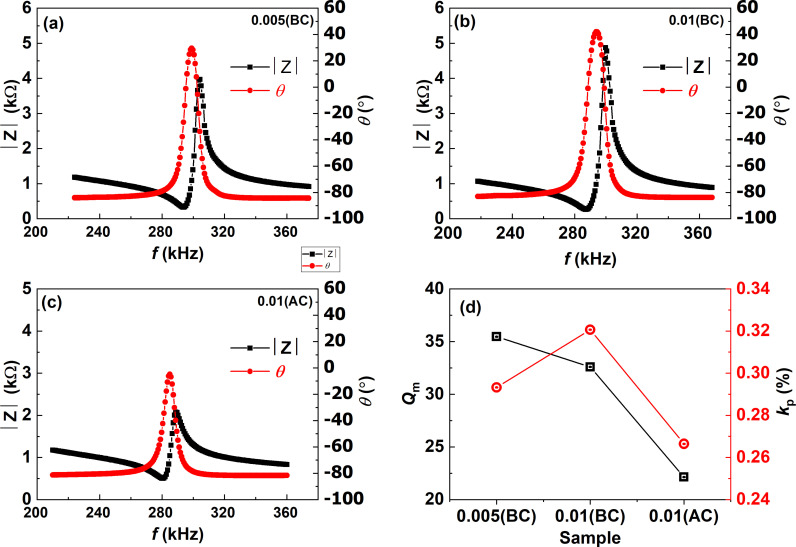
(a-c) Frequency characteristics of | Z | and *θ* of *x* =  0.005(BC), 0.01(BC), 0.01(AC), and (d) piezoelectric properties of *Q*_m_ and *K*_p_ as a function of Si concentration for BF30BT ceramics.

Fig 6(a-c) displays the frequency dependence impedance |Z| and phase angle (*θ*) of poled samples. According to the impedance spectra, the samples exhibit a resonance frequency of around (290–304) kHz. Fig 6(d) illustrates the mechanical quality factor *Q*_m_ and electromechanical coupling factor k_p_ calculated from their corresponding spectra. At resonance and anti-resonance frequencies, the impedance curves exhibit abrupt variations. These characteristics are consistent with observations for other hard piezoelectric ceramics [50]. The phase angle shift for the BF30BT ceramics was low, about 30.5°, but rose with increasing Si content to approach 50.7° for *x* =  0.005(BC)-0.01(BC). The higher *θ* value suggested that the substitution of Si^3+^ ions for Ti^4+^ ions resulted in a perfect poling state, which improved dielectric performance. The optimal poling condition is reached when *θ* approaches 90° in the frequency range between the resonance and anti-resonance frequencies. With increasing frequency, a perfect piezoelectric material exhibits a phase angle change from −90° to 90° during resonance and anti-resonance [50].

As the concentration of Si increased from 0.00 to 0.005(BC) wt%, the values of k_p_ increased dramatically from 0.295 to 0.325 then decreased to 0.27 at *x* =  0.01(AC) as can be seen in Fig 6(d). It can be explained that as Si_2_O_3_ is doped at *x* =  0.01(AC), the grain size decreases, the domain wall is pinched or clamped by more grain boundaries, and the domain is difficult to reverse by electric field, resulting in low k_p_ value. As *x* increases from 0.005(BC) to 0.01(BC), it can be seen that the k_p_ increases from 0.29 percent to 0.32 percent. For *x* =  0.01(BC), yields the highest k_p_ =  0.32% ascribing to the phase structure MPB [51]. For *x* =  0.005(BC), yields the highest *Q*_m_ (32.6) and a higher *Q*_m_ suggests suppression of domain wall motion. Because the acceptor doping creates oxygen vacancy or defect dipoles that can occupy energetically favored lattice locations. These anisotropic centers induce an internal bias field and pinning or clamping of the domain wall motion that leads to high *Q*_m_ values [52–54]. The piezoelectric strain performance under the applied field and *T*_C_ of this work were compared with other piezoelectric ceramics in Table 1. A large *d*_33_^ *^ ≈  750 pm/V under the 50 kV/cm with a high *T*_C_ ≈  450 °C of this work, are fascinating results for high-temperature device applications.

**Fig 7:**
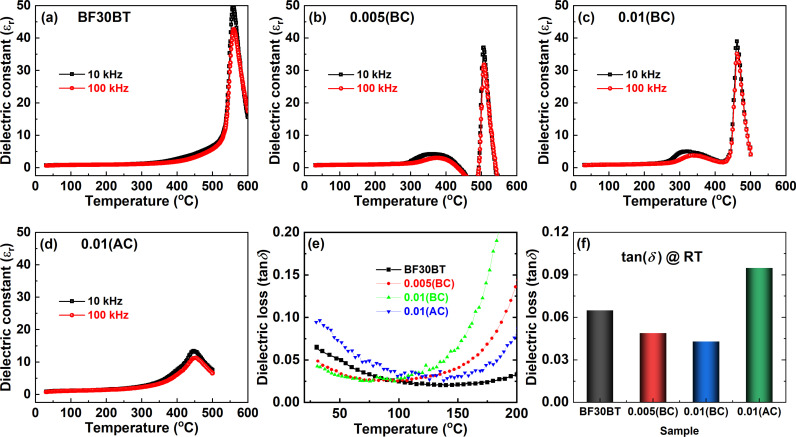
(a-d) Temperature-dependent dielectric constant (*ε*_r_) from room temperature to 600 °C, (e) tan*δ* from room temperature to 200 °C, and (f) room temperature tan*δ* for Si-doped BF30BT ceramics.

Fig 7, the temperature-dependent dielectric constant (*ε*_r_) for Si-doped BF30BT ceramics at 10 kHz and 100 kHz in the temperature range of 25°–600 °C. The pure BF30BT ceramic illustrates typical ferroelectric behavior and exhibits a very sharp peak with *T*_C_ ≈  560 °C [Fig 7(a)]. The Si-doping in BF30BT ceramics induces an additional dielectric peak near 350-450 °C as shown in Fig 7(b, c). This anomaly is related to the compositional heterogeneity or defect charges migration at high temperatures [5]. Both anomalies are seen on the dielectric constant curves, one at depolarization temperature (*T*_d_), and the other at the dielectric maximum temperature (*T*_max_). The Si fusion lowers *T*_d_ to ambient temperature but does not affect *T*_max_. In addition, the dielectric constant peak at *T*_max_ is reduced and flattened in ceramics with a larger Si content. Similar dielectric anomalies are seen in Mn-doped BF-BT ceramics [55]. However, the wide/broad transition peak can be observed for 0.01(AC) suggesting the relaxor ferroelectrics behavior [Fig 7(d)]. The weak peaks can be attributed to oxygen tilting or the presence of defects in these ceramics. With the addition of Si content, the maximum dielectric constant declines fast and the *T*_m_ peaks become exceedingly broad. Furthermore, the *T*_d_ peaks slowly disappear as the Si content increases, indicating that *T*_d_ has been relocated below room temperature. As disorder arises, the material’s structure becomes inhomogeneous, resulting in a better range of local conditions for the dipoles. This leads to a larger temperature range for dipole alignment, obscuring the dielectric constant peak. In classical ferroelectrics, peaks in the real component of the dielectric constant are sharp and do not move to higher temperatures as frequency increases because ferroelectric phase transition is well-defined and nearly frequency-independent. However, if the *T*_d_ peaks gradually disappear as the Si content increases, it means that *T*_d_ has been shifted below room temperature. The decrease in *T*_d_ caused by Si modification confirms the compositionally induced ferroelectric-relaxor transition. This behavior is typical of a relaxor, in which the peak broadens and changes due to the presence of high local disorder and weak dynamics in polar nanoregions.

As shown in Fig 7(e) for all compositions the dielectric losses gradually decrease up to ≈  100 °C and then substantially increase at the higher temperature. It also showed that the small amount of Si_2_O_3_ additive had little effect on the dielectric loss of BF30BT ceramics, making it appropriate for high-power applications. The room temperature dielectric losses of the pure BF30BT ceramic dramatically decrease from 0.065 to 0.043 for *x* =  0.01(BC) and again raised to tan*δ* ≈  0.095 for *x* =  0.01(AC) as shown in Fig 7(f). Tan losses are minor at 100 °C, but increase sharply at high temperatures, indicating an increase in DC conductivity. When the Si concentration of the BFBT was increased to *x* =  0.01(AC), the dielectric permittivity widened and the *T*_C_ fell down. The *T*_C_ is moved toward the lower temperature through the relation as *T*_C_ ≈  (K_L_ − K_S_)/B, where K_L_ is the long-range electrostatic force constant, K_S_ is the short-range harmonic force constant and B is the inharmonic coefficient [15]. The Si doping in BFBT ceramics causes local structure heterogeneity, which suppresses long-range ferroelectric order, as a result of which a chemically induced phase change from the regular ferroelectric phase to the relaxor phase occurs. Normally, hard ferroelectric ceramics exhibited small dielectric losses as compared to their counterpart of soft-ferroelectric ceramics [56]. Hence, the dielectric properties further support that Si-addition before calcination induces a hard ferroelectric effect while after calcination it creates a soft ferroelectric effect. Furthermore, this increase in tan*δ* at high temperatures is mainly associated with long-range space charge conduction [33].

## 4. Conclusion

The existing investigation involved the fabrication of lead-free Si-modified 0.7Bi_1.03_FeO_3_-0.3BaTiO_3_ piezoelectric ceramics by a conventional solid-state method, followed by thermal quenching. The XRD investigation reveals a structural phase transition caused by composition, going from a predominately rhombohedral to a mixed rhombohedral and tetragonal symmetry. The dielectric, ferroelectric and electric-strain properties of these doped BF30BT-based ceramics were studied. The current investigation shows the comparative study of Si addition before, and after calcination, where the Si-acceptor doping induces hard-ferroelectric behavior that improves the electromechanical performance. A large strain and dynamic piezoelectric coefficient *d*_33_ * ~  750 pm/V were achieved simultaneously in a single critical composition at *x* =  0.01(BC). A new step forward in piezoelectric material exploration and actuation has been made possible by the outstanding electromechanical properties of lead-free ceramics. The currently investigated ceramics show significant progress in lead-free piezoelectric actuators and sensors for high-temperature applications.

## Supporting information

S1 File **S1 Fig**. This is the schematic figure. The potential applications of ultrahigh electrostrain piezoceramic materials and their preferred devices. **S2 Fig**. This is the schematic figure. The functional properties of lead-free piezoelectric BiFeO3-BaTiO3 ceramic and desired parameters. **S3 Fig**. Data set for Fig. 3 (a) The X-ray diffraction patterns in the suggested range of 2*θ* =  10° − 60°, (b) magnified view for (111) peak at 2*θ* =  38° − 40° and (200) peak at 2*θ* =  38° − 40°, and (c, d) XRD Rietveld refinements patterns of *x* =  0.00(BC), 0.01(BC) samples for Si-modified BF30BT solid solutions. **S4 Fig.** Data set for Fig. 4 (a-d) Ferroelectric polarization (*P*−*E*) hysteresis loops for Si-doped BF30BT ceramics. **S5 Fig.** Data set for Fig. 5 (a, b) Butterfly bipolar strain-electric field (*S*−*E*) curves recorded under an applied electric field of 50 kV/cm, (c) maximum strain (*S*_max_), and (d) *d*_33_, *d*_33_^*^ of BF30BT ceramic as a function of Si^3+^ concentration. (e) Nd-doped BF30BT [23], BF40BT-BiM_2/3_Nb_1/3_O_3_ (BF40BT-BMN)[57], Sm-doped BF30BT (BSmF30BT) [24], Nb-doped BF33BT (BF33BTNb) [1], BF35BT [58], Zr-doped BF33BT (BF33BZrT) [14], BFBT-BiScO_3_ [28], Zr-doped BF36BT [30], LiNbO3-BF33BT (BF33BT-LN) [31], Ba-site Sm-doped BF33BT (BF33BSmT) [59], Bi-site BF33BT (BSmF33BT) [60], PZT and KNN-textured [8]. S6 Fig. Data set for Fig 6(a-c) Frequency characteristics of | Z | and *θ* of *x* =  0.005(BC), 0.01(BC), 0.01(AC), and (d) piezoelectric properties of *Q*_m_ and *K*_p_ as a function of Si concentration for BF30BT ceramics. **S7 Fig.** Data set for Fig. 7 Fig. 7: (a-d) Temperature-dependent dielectric constant (*ε*_r_) from room temperature to 600 °C, (e) tan*δ* from room temperature to 200 °C, and (f) room temperature tan*δ* for Si-doped BF30BT ceramics.
(XLSX)
